# 脂质体与外泌体在药物递送和生物标志物筛选中的研究进展

**DOI:** 10.3724/SP.J.1123.2024.08012

**Published:** 2025-05-08

**Authors:** Yating SU, Xiaohong QIAN, Weijie QIN

**Affiliations:** 医学蛋白质组全国重点实验室, 国家蛋白质科学中心(北京), 北京蛋白质组研究中心, 军事科学院军事医学研究院, 北京 102206; State Key Laboratory of Medical Proteomics, National Center for Protein Sciences (Beijing), Beijing Proteome Research Center, Beijing Institute of Lifeomics, Beijing 102206, China

**Keywords:** 脂质体, 外泌体, 囊泡, 富集和分离, 药物递送, 生物标志物筛选, 综述, liposomes, exosomes, vesicles, enrichment and separation, drug delivery, biomarker screening, review

## Abstract

囊泡分为人工囊泡和天然囊泡。其中,人工囊泡的一种代表性形式是脂质体,它们是由人工合成的,由一个或多个磷脂双分子层构成的球形脂质结构。脂质体的生物相容性和生物利用度较高,稳定性好且易于合成,是靶向药物递送系统(DDS)中常用的纳米载体之一,使用频率较高。作为一种天然囊泡,细胞外囊泡(EVs)是由细胞主动分泌的一种具有膜结构的微小囊泡,内含核酸、蛋白质及脂类等多种成分,是细胞间通信的重要媒介。外泌体是EVs中的最小亚型,直径为30~100 nm,内含独特的生物分子,这些分子可被视为亲代细胞的“指纹”。在病理状态下,外泌体的含量会发生变化,因此被视为诊断疾病的潜在生物标志物。近年来,脂质体与外泌体在药物递送和生物标志物筛选领域日益受到关注。本文介绍了脂质体的制备技术及外泌体的富集分离技术,并深入探讨了它们在药物递送及生物标志物筛选方面的研究进展。最后,文章提出了脂质体与外泌体在临床应用中所面临的挑战。

## Abstract (en)

Vesicles, are categorized as artificial (i.e., liposomes) or natural (i.e., extracellular vesicles (EVs)) and play significant roles in drug-delivery and biomarker-screening applications. Liposomes, as a representative form of artificial vesicle, are spherical lipid structures composed of one or more artificially synthesized phospholipid bilayers. Liposomes are highly biocompatible and bioavailable, very stable, and easily synthesized; hence, they are among the most commonly used and frequently applied nanocarriers in targeted drug-delivery systems (DDS). EVs are natural small membrane-bound vesicles actively secreted by cells and contain a variety of components, including nucleic acids, proteins, and lipids. They also serve as important mediators of intercellular communication. As the smallest EV subtype, with diameters of only 30-100 nm, exosomes contain unique biomolecules that are considered to be the fingerprints of the parent cells. In the pathological state, the content of exosomes will change; consequently, exosomes are potential disease-diagnosis biomarkers. Recent clinical trials have shown that exosomes are ideal nanocarriers in targeted drug-delivery therapies for a variety of diseases. Compared with traditional artificial liposomal carriers, exosomes display unique advantages and provide the DDS field with new possibilities. Liposomes and exosomes are receiving increasing levels of attention in the drug-delivery and biomarker-screening fields. This article introduces techniques for the preparation of liposomes, and the enrichment and separation of exosomes, and delves into research progress on their use in drug-delivery and biomarker-screening applications. Finally, challenges facing the use of liposomes and exosomes in clinical applications are discussed.

为了最大限度地提升治疗效果,同时降低副作用等不良反应的风险,药物递送系统(DDS)的发展已成为广泛研究的焦点^[1]^。作为DDS的主体,脂质体和外泌体等纳米载体凭借低免疫原性、良好的生物相容性和高生物利用度等优势,已在临床领域得到广泛应用,包括免疫疾病、神经系统疾病、血液系统疾病和癌症等多种疾病的治疗研究^[2,3]^。脂质体的发现可以溯源到20世纪60年代,早在1965年,剑桥大学的Bangham等^[4]^就提出了脂质体可作为生物膜模型的观点,实验中揭示了脂质体的生物降解性和生物相容性,为脂质体作为药物载体奠定了基础。1971年,英国科学家Gregoriadis和Ryman^[5]^首次报道了脂质体在DDS中的应用,并对此进行了相关研究。自此以后,脂质体作为一种广泛应用的药物载体,成为生物医药纳米材料领域的研究热点。细胞外囊泡(extracellular vesicles, EVs)最早由Wolf教授于1967年在血浆中发现^[6]^。直到1987年,Johnstone教授才提出了外泌体的概念^[7]^。外泌体是EVs中的最小亚型,直径为30~100 nm,内含独特的生物分子,这些分子可被视为亲代细胞的“指纹”。在病理状态下,外泌体的含量会发生变化,因此被视为诊断疾病的潜在生物标志物^[8]^。近年来的临床试验也表明,外泌体作为一种理想的纳米载体,可用于各种疾病的靶向药物递送治疗^[9]^。与传统的人工载体脂质体相比,外泌体具有独特的优势,为DDS领域带来了新的可能。

## 1 脂质体的制备技术及外泌体的富集分离技术

在研究脂质体和外泌体之前,首先要对二者进行制备和富集分离。稳定的制备方法和高特异性的富集分析技术有助于更好地对这二者展开深入研究。当前,存在多种富集分离方法,这些方法对脂质体和外泌体的浓度、体积、形态及粒径大小各有不同的影响。因此,选择合适的富集分离方法以确保获得高纯度的脂质体和外泌体,对于推动其后续研究具有重大意义。

### 1.1 脂质体的制备技术

#### 1.1.1 薄膜分散法

薄膜分散法是最早用于制备脂质体的技术,也是目前应用最广泛的技术^[4]^。该技术首先将磷脂和胆固醇溶解于有机相中,再通过蒸发过程去除有机溶剂,从而形成脂膜;随后,将脂膜与水介质一起进行分散或水合,其中分散或水合过程应在搅拌或超声条件下进行,以确保附着在旋转蒸发烧瓶内壁的薄膜能够有效分离并转化为封闭的球形结构。若需包裹药物或其他成分,则可根据被包裹物质的性质,在形成薄膜前将其与脂类混合(适用于亲脂性化合物)或在水介质中加入(适用于亲水性化合物)。薄膜分散法最大的优点是操作简单,并且即使在处理少量化合物时也具有很高的重现性,特别适用于小剂量亲脂性成分的包埋。然而,该方法最大的缺点是包封率低,难以去除有机溶剂,以及易产生大小不一的多层囊泡脂质体(multilamellar vesicles, MLVs)^[4,10]^。

薄膜分散法已被许多研究者应用。Shen等^[11]^采用该方法制备了盐酸小檗碱负载的脂质体凝胶,旨在探究其抗氧化性能及对小鼠湿疹模型的治疗效果。Wang等^[12]^先通过薄膜分散法制备脂质体,随后利用谷胱甘肽-明胶(GSH-GG)的离子响应特性在脂质体外层构建凝胶,成功合成了可用于封装阿霉素(DOX)的钙交联脂质体凝胶。与传统的脂质体相比,该脂质体凝胶展现出了长期释放药物的优势,有助于增强肿瘤部位的药物累积,从而提升抗肿瘤疗效。

#### 1.1.2 溶剂注入法

溶剂注入法是指先将脂类溶解于有机溶剂中,随后将脂溶液注入水介质中,以此形成脂质体。溶剂注入法分为乙醇注入法和乙醚注入法,其中乙醇注入法于1937年被首次提出^[13]^。乙醇注入法的优势在于操作简便、快捷,通过将含有脂质的乙醇溶液一步注入水中,即可获得粒度分布较小的脂质体,无需额外的挤压或超声等操作^[14]^。然而,该方法也存在一些局限性,具体包括:对亲水性化合物的包封率较低,脂类在乙醇中的溶解度有限,以及生物活性大分子容易在乙醇环境中失去活性^[15]^。Santo等^[16]^采用乙醇注入法制备脂质体,并结合超临界流体萃取技术来去除脂质体溶液中残留的乙醇。在超临界萃取过程中,通过使用荧光素溶液处理脂质体,成功实现了90%的包封率。

与乙醇注入法相比,乙醚注入法由于乙醚不溶于水,能够制备出更高浓度的脂质体。然而,该方法在注射过程中要求在不同温度条件下操作乙醚与水相,这可能会影响乙醚对某些化合物的包封率,同时导致所形成脂质体的形状极不均匀^[17⇓-19]^。除了用于制备脂质体,近年来有学者利用乙醚注入法成功制备了免疫刺激复合物(ISCOM)。该研究表明,乙醚注入法有效克服了现有ISCOM制备方法的诸多局限性,并被视为一种具有可扩展性和连续性的ISCOM制备新方法。^[20]^。

#### 1.1.3 逆相蒸发法

逆相蒸发法最初由Szoka和Papahadjopoulos共同提出^[21]^,其基于反胶束的形成原理。该方法通过将脂类溶解于有机溶剂中,随后加入少量水相,并对混合溶液进行超声处理以形成倒置胶束。逆相蒸发法能够包封药物、蛋白质、核酸以及其他生化试剂。然而,与其他方法相比,逆相蒸发法的缺点在于被包封的化合物会与有机溶剂发生接触,因此该方法不适用于多肽等分子的包封^[17,21]^。Das等^[22]^通过逆相蒸发法将产黄青霉SNP5的脂质双层重组为脂质体,并发现这种脂质体的热稳定性达到了最大值。Handa等^[23]^对逆相蒸发法进行了改良,采用改良后的方法制备脂质体,可将脂质体的药物包封率提升至约80%。

#### 1.1.4 复乳法

复乳法的具体步骤如下:首先,将少量水相加入到含有较多量磷脂的不相容有机溶剂中,形成油包水(W/O)型乳状液;接着,将此乳状液加入到水相中,形成复乳;最后,通过去除有机溶剂,得到脂质体溶液。复乳法特别适用于包封水溶性药物,并能提供较高的药物包封率^[17]^。Li等^[24]^分别采用复乳法和改进后的复乳-动态高压微流态化法,制备了同时含有亲水性药物(维生素C)和疏水性药物(中链脂肪酸)的复合脂质体。结果表明,采用复乳-动态高压微流态化法制备出的复合脂质体不仅具有较高的包封率,且在4 ℃条件下保存时展现出了良好的稳定性。

#### 1.1.5 洗涤剂耗尽法

洗涤剂耗尽法是一种相对温和的方法,能够生产出多种类型的囊泡以及高度均一的脂质体。该方法基于洗涤剂-脂质胶束体系,通过逐步去除洗涤剂,最终制得脂质体。该方法的优势是能够形成单层囊泡结构的脂质体,这些脂质体具有较大的包封量且大小均匀^[25]^。然而,该方法的制备过程和富集过程非常耗时,并且在去除洗涤剂的同时可能会连带去除其他小分子亲水性化合物^[17]^。在研究该方法制备脂质体的影响因素时,Lasch等^[26]^发现,洗涤剂在去除过程中形成脂质体的效率取决于其自身的临界胶束浓度(CMC)以及对脂质体膜的亲和力。

#### 1.1.6 其他方法

随着研究的不断深入,脂质体在实验室规模的生产已趋于成熟。然而,脂质体的工业化大规模生产仍面临诸多挑战。因此,近年来越来越多的脂质体制备方法被开发出来,其中基于超临界的方法发展尤为迅速,主要包括超临界反溶剂法^[27,28]^、超临界反相蒸发法^[29,30]^、超临界溶液快速膨胀法^[31⇓⇓⇓⇓-36]^和超临界辅助脂质体形成法^[37,38]^等。此外,Laouini等^[39]^结合膜接触器技术与中空纤维模块,创新性地开发出了一种脂质体制备新方法。该方法凭借膜接触器的高效传质性能,结合中空纤维模块的精密结构设计,实现了脂质体的高效制备。通过将脂质溶液流经中空纤维膜接触器,使其与水相充分接触,进而形成均匀的脂质体。Hirsch等^[40]^利用双不对称离心技术,迅速制备出了无菌且包封率较高的脂质体;Wang等^[41]^采用冷冻干燥液法制备出了重现性强、稳定性好、平均粒径小(约100 nm)和包封率高(钙黄绿素为87%,氟比洛芬为93%, 5-氟尿嘧啶为19%)的脂质体;Abrams等^[42]^利用微流控法制备了包封有小干扰RNA(siRNA)的脂质体,使得小鼠肝脏中的靶向信使RNA(mRNA)沉默效率超过80%。

### 1.2 外泌体的富集分离技术

#### 1.2.1 离心法

离心法可分为差速超速离心法和密度梯度离心法,其中差速超速离心法应用最为广泛,且被视为外泌体富集与分离的金标准方法^[43]^。该方法的操作流程通常包含3个阶段:(1)低速离心(3×10^2^ g),以去除细胞和凋亡碎片;(2)加速离心(2×10^2^ g),以去除较大的EVs; (3)超速离心(>1×10^5^ g),以沉淀外泌体,并通过磷酸盐缓冲盐溶液洗涤来去除杂质。该方法利用高速离心力将外泌体从样品中提取分离出来,适用于处理沉降系数存在显著差异的大量样品。然而,该方法无法分离与外泌体具有相似生物物理特性的脂蛋白。并且,该方法耗时较长且提取效率相对较低,外泌体的产量和纯度易受到离心时间、离心力强度、转子类型等多种因素的影响^[44]^。此外,多次离心处理可能会对外泌体的结构造成损伤,不利于后续的蛋白组学分析等研究^[45]^。

密度梯度离心法将样品置于密度梯度介质(如碘克沙醇或蔗糖)的顶部,随后进行长时间的离心处理。这一过程能够将细胞外成分(如外泌体、凋亡囊泡和蛋白质聚集体等)分离成具有不同浮力密度的成分^[46,47]^。具体而言,蛋白质聚集体会主要集中在离心管的底部,而外泌体则保留在浮力密度为1.1~1.2 g/mL的介质层中^[48]^。该方法的分离效果好,可获得纯度较高的外泌体,但该方法耗时较长,且所获得的外泌体含量较少^[49,50]^。因此,为了提高外泌体的纯度并减少耗时,可以联合使用密度梯度离心法与差速超速离心法进行提取。

#### 1.2.2 尺寸排阻色谱法

尺寸排阻色谱法是一种色谱分离技术,它根据分子尺寸或在某些特定情况下依据分子质量大小来分离溶液中的分子,是分离外泌体最常用的技术手段之一。该技术通常采用多孔凝胶颗粒(如葡聚糖聚合物、琼脂糖、聚丙烯酰胺等)作为填充物,当样品颗粒流经尺寸排阻色谱柱时,较小的颗粒能够进入凝胶内部的小孔,而较大的颗粒则无法进入,只能在凝胶颗粒间的空隙中穿行,因此较大的颗粒会先被洗脱出来^[51]^。尺寸排阻色谱法具有产率高、重现性好和操作简单等优点,并且该方法不会影响外泌体的生物学特性,具备处理多种类型和大量样品的能力^[52]^。Guan等^[53]^利用尺寸排阻色谱法成功分离了尿液中的外泌体,并首次对尿液外泌体进行了尺寸依赖性的亚蛋白质组分析。Zheng等^[54]^开发了一种连续尺寸排阻色谱法,该方法能够高效地从人血浆中分离出外泌体。Gao等^[55]^开发了一种能够从人血清中快速分离外泌体的新策略,该策略基于TiO_2_与外泌体磷脂双层上磷酸基团之间的特异性相互作用,并结合尺寸排阻色谱法,能够获得较高的纯度和较高的回收率(93.4%)。目前,基于尺寸排阻色谱法原理的纯化技术,如IZON^®^ qEV柱^[56]^、Sepharose^®^ CL-2B柱^[57]^和Sephacryl^®^ S-400柱^[58]^,已经实现了商业化应用。与传统的超速差速离心法相比,采用尺寸排阻色谱法富集外泌体时,能够更有效地分离游离的微小RNA(miRNA)与外泌体内部的miRNA^[59]^。然而,该方法无法同时对多个样本进行处理,并且需要特定的设备和填料^[52]^。

#### 1.2.3 过滤法

过滤法是富集和分离外泌体最简便的方法之一,其基本原理类似于传统的膜过滤技术,通过采用不同截留分子质量的超滤膜对样品进行选择性分离,从而获取外泌体^[60]^。目前,过滤法主要分为死端过滤和切向流过滤两种类型。死端过滤法适用于处理体积较小的样本,而对于体积较大的样本,则应选择切向流过滤法^[60]^。切向流过滤法的分离速度快,能够直接从大量样本中提取外泌体成分。但该方法对外泌体的提取纯度不高,并且膜污染问题容易导致样品损失^[61]^。Haraszti等^[62]^发现,与差速超速离心法相比,采用切向流过滤法富集间充质干细胞外泌体时,其产量可提高140倍。Lee等^[63]^发现,利用切向流过滤法可以重复且大规模地生产出源自脂肪组织间充质干细胞的外泌体。

#### 1.2.4 聚合沉淀法

早期研究已表明,聚合物沉淀法可用于富集和提取病毒颗粒,如今该方法也可应用于外泌体的富集与分离^[64]^。该方法依据尺寸和密度进行分离,主要包括鱼精蛋白沉淀法^[65]^、乙酸钠沉淀法^[66]^、有机溶剂沉淀法^[67]^以及亲水多聚物沉淀法^[68]^等4类常见方法。在亲水多聚物沉淀法中,聚乙二醇(PEG)常被用作介质,通过降低外泌体在离心过程中的溶解度来实现外泌体的收集。该方法的效率高,适用于大剂量样品的处理^[69]^。Ye等^[70]^发现,与离心法相比,PEG沉淀法能够在更短的时间内从人脐带血血浆中富集并分离出更多的外泌体。然而,PEG的用量对外泌体的富集效率有所影响。Shieh等^[71]^基于PEG,优化了口腔鳞状细胞癌(OSCC)衍生外泌体的提取方法。研究分别使用不同质量浓度(8%、10%和12%)的PEG从培养基中分离外泌体,并将所获外泌体的纯度与超速差速离心法进行比较,结果表明,8%的PEG是OSCC衍生外泌体分离及其下游应用的理想选择。

#### 1.2.5 基于免疫亲和法

免疫亲和法是一种传统且常用的外泌体分离技术,它基于抗原-抗体相互作用原理,通过一方(抗原或抗体)作为配基来亲和吸附另一方,从而实现外泌体的富集与分离^[72]^。外泌体表面富含特殊的膜蛋白,如CD9、CD63、CD81、CD82、膜联蛋白和上皮细胞黏附分子等,这些蛋白能与固定在不同基质上的同源抗体特异性结合,进而达到分离和富集外泌体的目的^[73]^。目前,依据所使用抗体包被的基质类型,免疫亲和法可进一步细分为酶联免疫吸附分离法、磁珠分离法以及芯片法等^[74]^。

酶联免疫吸附法通过在微孔板上包被外泌体特异性抗体,可从各种生物体液中有效富集外泌体,并且该方法还能够对富集后的外泌体进行定量分析^[75]^。相比之下,磁珠分离法因磁珠的稳定性差、富集效率低下以及可能对外泌体生物活性产生不良影响而面临应用上的局限^[76]^。因此,改进免疫亲和材料成为提升该方法性能的关键途径。Zhang等^[77]^设计了一种免疫亲和片,该亲和片通过将Tim4抗体接枝到具有强亲水性的金属有机框架上制成,能够在中性条件下高效地富集外泌体,同时保持良好的外泌体活性。此外,Guo等^[78]^开发了一种新型磁性纳米颗粒,利用链霉亲和素突变体(strep-tactin)与strep-tag Ⅱ(由8个氨基酸(WSHPQFEK)组成的小标签)之间的特异性且可逆的识别作用,将捕获抗体修饰到磁性纳米颗粒上,构建了基于strep-tag Ⅱ的免疫磁性分离(SIMI)系统。该方法能够在短时间内高效富集大量外泌体。与被视为金标准的差速超速离心法相比,SIMI系统不仅从人胚肾细胞(293T)培养基中多收获了近59%的外泌体,而且分离过程耗时更短,所得外泌体的纯度也更高。

芯片法是一种融合了免疫亲和法与微流控技术的方法。芯片法具有样本需求量少、回收率高以及所得外泌体纯度高的优点^[79]^。因此,近年来众多学者不断致力于该方法的创新与优化。Zhao等^[80]^设计了一种ExoSearch外泌体分离芯片,用于研究卵巢产生的外泌体。该芯片配备两个流体入口:一个用于注入含有特异性抗体包裹的免疫磁珠溶液,另一个则用于注入待检测的血浆样品。此外,该芯片能够定量分离和富集不同制备量(10 μL~10 mL)的血浆外泌体。Chen等^[81]^开发了一种能够从血液样本的血清和培养基中富集外泌体的新方法,该方法通过在微流控装置通道表面涂覆一层生物素化的CD63抗体,来捕获含有CD63抗原的外泌体。同样基于这一原理,Kanwar等^[82]^研发了Exochip芯片,用于富集胰腺癌患者血清中的外泌体。Liu等^[83]^设计了一种ExoTIC芯片,旨在简化外泌体的提取过程。与传统的外泌体分离方法相比,ExoTIC芯片具有从临床样本以及小样本量中高产量提取外泌体的优势。此外,该芯片还适用于疾病诊断中的外泌体即时检测。

#### 1.2.6 基于微流控技术

除了上述将微流控技术与免疫亲和相结合的方法外,基于微流控技术富集外泌体还存在其他多种途径。其中一种方法是将微流控技术与声学相结合,即声流体法,它利用声波原理来分离微流体中不同大小的颗粒。Lee等^[84]^基于该原理,开发了一种能够分离外泌体的声纳米过滤系统微流控芯片,并成功地从细胞培养液中提取出了微囊泡和外泌体。此外,还有一种基于介电泳分离外泌体的方法。Ibsen等^[85]^设计了一种交流电动微阵列芯片装置,并利用该装置成功地从血浆样品中富集到胶质母细胞瘤的外泌体。除此之外,外泌体的分离还可以基于微流体动力学特性来实现。Wunsch等^[86]^基于确定性横向位移原理,成功地从人尿中富集到直径为60~70 nm的外泌体。Liu等^[87]^利用黏弹性微流体的特性,以连续、尺寸依赖且无标记的方式,直接从细胞培养基或血清中富集到外泌体。此外,随着纳米材料的不断进步,也可将新型纳米材料与微流控技术相结合,用于外泌体的富集与分离。有研究^[88]^报道,通过在纳米线表面修饰CD9、CD63和CD81等抗体,可以有效地富集样本中携带了这3种膜蛋白的外泌体,随后经过二硫苏糖醇的处理,即可获得外泌体颗粒。

## 2 脂质体的药物递送应用

DDS通过增加药物在靶细胞中的浓度和停留时间来最大限度地减少副作用,并提升疗效^[89]^。DDS利用纳米载体将活性药物递送至作用位点,以增强游离药物的药理作用,同时通过优化药物的药代动力学特性和生物分布,来改善其原有的不良特征^[90,91]^。近年来,纳米材料作为DDS已引起了广泛关注。其中,纳米载体是最常用的介导靶向药物递送的药物载体系统^[92]^,其尺寸通常介于几纳米至几百纳米之间^[93]^。目前,制造纳米颗粒的材料多种多样,包括天然材料、有机及无机材料,如脂质、聚合物、陶瓷以及金属^[93]^等。脂质类材料又可细分为胶束和脂质体等纳米颗粒^[94⇓-96]^。被包封的药物主要通过物理相互作用(如包埋、表面附着或封装等方式)被掺入这些纳米颗粒中^[97]^。

尽管纳米药物领域取得了飞速发展,但大多数基于纳米颗粒的DDS仍面临负载能力不足和靶点特异性有限的挑战^[98]^。因此,当前的研究趋势是深入探索并设计出具有高负载能力和可调节靶向性的纳米载体^[99]^。脂质体作为靶向DDS中应用最广泛的纳米载体,已成功开发出多种制剂并应用于临床治疗,如抗肿瘤治疗、抗真菌疗法及疫苗等领域^[100]^。脂质体在生物医学领域的应用见图1。

**图1:**
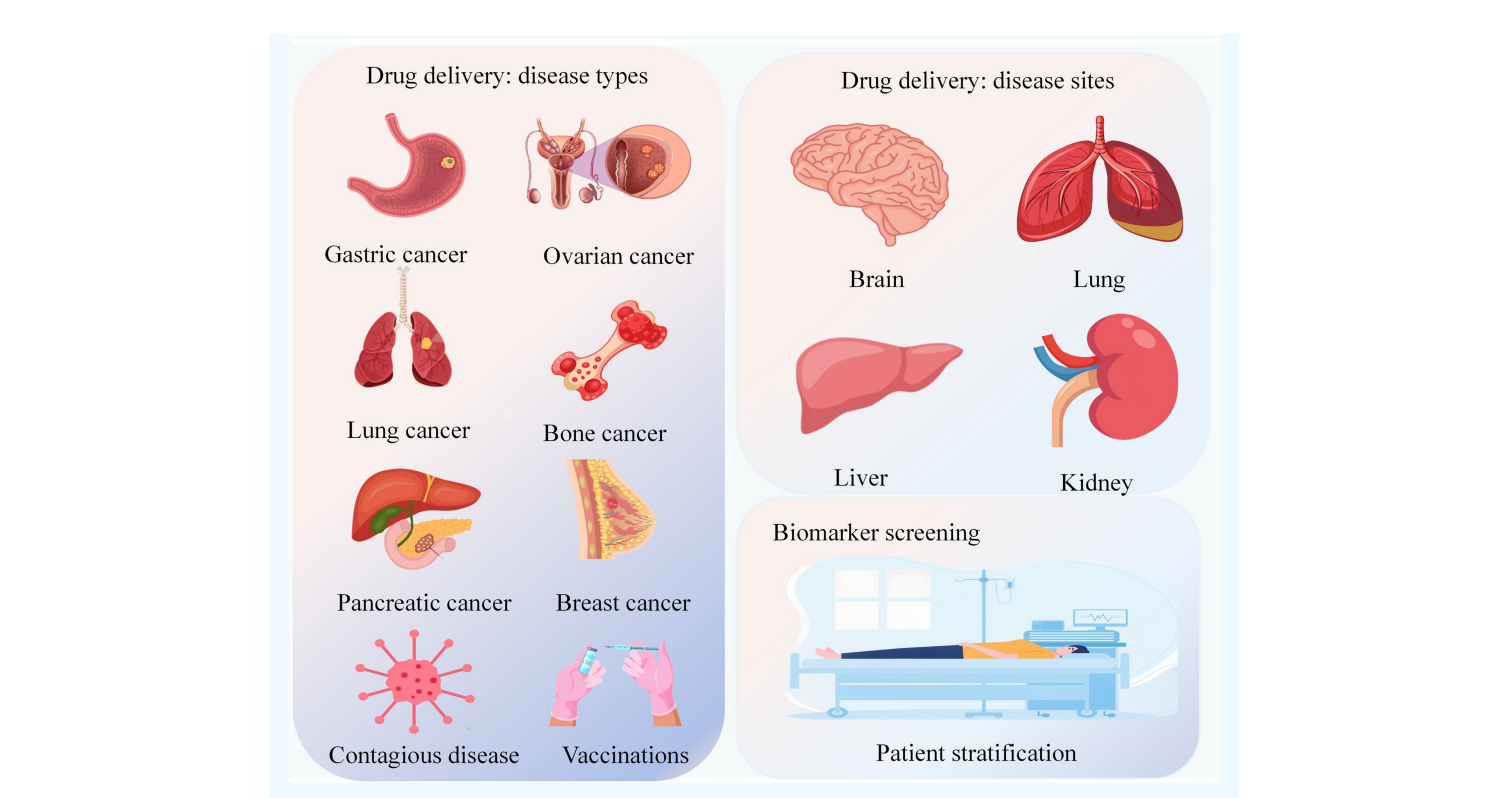
脂质体的生物医学应用

### 2.1 基于疾病类型的药物递送应用

#### 2.1.1 肿瘤靶向的应用

肿瘤治疗药物存在诸多弊端,它们往往会同时伤害肿瘤细胞与健康细胞。相比之下,纳米颗粒介导的靶向DDS通过增强渗透性和滞留作用(EPR),促进了药物在肿瘤部位的积聚,提高了药物的溶解度、生物利用度和治疗效果,同时降低了游离药物浓度,减少了不良反应。此外,通过在脂质体表面涂覆聚合物(如PEG),不仅能延长脂质体在血液中的循环时间,还能有效隐藏脂质体,避免被网状内皮系统摄取^[101]^。PEG具有多重功效,它不仅能遮蔽脂质体表面的电荷,还能增强脂质体与血液成分之间的排斥力。此外,PEG能提升脂质体表面的亲水性,并在其表面形成一层聚合物保护层,这层保护层不仅增强了脂质体的稳定性,还降低了其在血液循环中被迅速清除的风险^[102]^。Doxil^®^是首个获得美国食品药品监督管理局(FDA)批准、用于治疗多种癌症的PEG化脂质体DOX。它通过创新的pH梯度主动负载技术和PEG化隐形脂质体的应用,为其他多种脂质体制剂的临床应用开辟了新的道路^[103]^。此外,脂质体表面的组成和电荷是影响其靶向性的其他重要因素,可通过在脂质体表面缀合特定的靶向分子(如适配体、凝集素、转铁蛋白(TF)、叶酸和抗体等)来提高其靶向性^[104]^。表1汇总了部分用于肿瘤治疗的经典脂质体产品。

**表1:** 部分用于肿瘤治疗的脂质体产品

Liposome product	Ingredient	Indications	Ref.
Doxil^®^	adriamycin	breast cancer, ovarian cancer, and multiple myeloma	[103]
DaunoXome^®^	erythromycin	AIDS-infected Kaposi’s tumor	[105]
Myocet^®^	adriamycin	metastatic breast cancer	[106]
Onivyde^TM^	irinotecan hydrochloride	metastatic pancreatic cancer	[107]
Mepact^®^	mifamurtide	non-metastatic osteosarcoma	[108]
Lipo-Dox	adriamycin	breast cancer, ovarian cancer, and Kaposi’s sarcoma	[109]
Lipusu	paclitaxel	stomach, ovarian and lung cancer	[110]

AIDS: acquired immune deficiency syndrome.

#### 2.1.2 传染病靶向的应用

传染病包括疟疾、结核病、艾滋病、霍乱等多种疾病,它们主要由细菌、病毒、真菌等微生物引起。治疗传染病的主要方法是通过使用抗菌化合物来抑制或中断这些微生物的重要细胞周期过程,从而达到杀灭它们的目的^[111]^。然而,治疗过程中常会遇到耐药性问题,因此需要采用靶向给药策略来应对感染性疾病。近年来,基于脂质体的靶向DDS在这类疾病中发挥了重要的作用。疟疾作为一种广泛传播的传染病,其病原体包括疟原虫属的4种寄生原生动物(恶性疟原虫、间日疟原虫、三日疟原虫和卵形疟原虫)。鉴于当前疟疾治疗中存在的副作用和耐药性问题,开发针对疟疾的靶向治疗药物方法尤为迫切。Marques等^[112]^研发了一种新型脂质体,该脂质体通过共价键结合肝素,实现了对疟原虫感染的红细胞(pRBC)的靶向递送功能。Pandey等^[113]^研制了包含利福平、异烟肼和吡嗪酰胺组合的雾化固体脂质颗粒(SLN),用于对感染了结核分枝杆菌的豚鼠支气管肺泡区域进行药物输送。结果表明,该SLN成功实现了从肺部和脾脏中完全清除结核杆菌的效果。有研究小组采用了沙奎那韦的脂质体递送系统,该研究证实,通过脂质体的包裹,能够提升沙奎那韦在巨噬细胞中的内化效率;与此同时,该脂质体递送系统可降低在高浓度使用时对细胞的毒性作用^[114]^。当前,艾滋病治疗面临的最大挑战之一是治疗过程中出现抗逆转录病毒药物的多药耐药性(MDR),这直接导致了不佳的临床结果^[115]^。基于纳米颗粒的抗逆转录病毒药物靶向递送策略已被广泛研究,不同研究团队针对不同细胞和治疗目标进行探究。Wu等^[116]^开发了一种叠氮胸苷棕榈酸酯(AZTP)的半乳糖基化脂质体,用于将叠氮胸苷(AZT)主动靶向至肝细胞。Garg等^[117]^研究了一种半乳糖基化脂质体,该脂质体能够装载AZT,并针对巨噬细胞上存在的凝集素受体进行靶向。

### 2.2 基于疫苗的药物递送应用

#### 2.2.1 脂质体疫苗的应用

疫苗是预防传染病最具成本效益的手段。在疫苗研发过程中,激发先天性和适应性免疫反应的能力至关重要。为了引发针对抗原的充分免疫反应,选择合适的免疫刺激分子(如佐剂)以及高效的递送平台至关重要。纳米颗粒疫苗递送系统不仅有助于延长疫苗分子与抗原呈递细胞(APC)的接触时间,其还可以与APC产生相互作用并自行触发免疫反应^[118]^。1974年,脂质体首次被报道用作疫苗佐剂和递送平台^[119]^。脂质体具有灵活性和多功能性,其携带的疫苗所诱导的免疫刺激可通过多种因素进行调控,这些因素包括脂质体的组成、尺寸、同质性、电荷,以及抗原或佐剂在脂质体中的位置^[120]^。Carstens等^[121]^研究了两种尺寸(500 nm和140 nm)的阳离子脂质体,用于封装含有质粒DNA(pDNA)的卵清蛋白(OVA),旨在将OVA抗原主动靶向至肝细胞。研究结果显示,在较大的脂质体尺寸下,pDNA疫苗展现出了最强的保留能力。此外,虽然添加PEG涂层能够增强淋巴引流,但并未对免疫反应产生改善作用。

当前,随着技术的不断进步和对免疫系统认识的日益加深,脂质体作为疫苗递送系统的应用正迅速发展。它们已被实践用于递送多种抗原(包括用于结核病疫苗的Ag85B-ESAT-6(H1抗原)和Ag85B-ESAT-6Rv2660c(H56抗原)^[122,123]^、甲型肝炎抗原(Epaxal)以及甲型肝炎病毒疫苗和流感病毒疫苗中的Inflexal V抗原(来自灭活流感的血凝素和神经氨酸酶)^[124]^),以预防不同的疾病。

#### 2.2.2 mRNA脂质体疫苗的应用

1961年,生物学家Brenner等^[125]^利用感染了噬菌体的细菌成功证明了mRNA的存在,并揭示了mRNA传递遗传信息的机制。1989年,Malone等^[126]^提出了将mRNA作为药物的构想,并成功实现了使用阳离子脂质体包裹mRNA,进而将其转染至真核细胞中进行表达。mRNA因其亲水性和负电荷等理化特性,虽被认定是安全的,但也因此难以自发穿透细胞膜,导致其通过质膜的被动扩散效率低下^[127]^。此外,游离状态的mRNA在体内会迅速降解,较短的半衰期限制了其达到预期功效的能力^[128]^。鉴于此,开发一种递送载体以提高mRNA到达靶位点的效率尤为重要。脂质体相关的纳米技术已被证实能有效保护mRNA免受降解,延长其循环半衰期,并增强疫苗接种的效果。由于2019年严重急性呼吸综合征冠状病毒2(SARS-CoV-2)引发的呼吸道疾病在全球流行,针对SARS-CoV-2的治疗和预防方案一直在加速开发。在此背景下,mRNA疫苗成为首批进入临床试验的先锋之一。2020年,FDA授予了一个基于mRNA技术的新型冠状病毒疫苗(即BNT162b2 mRNA疫苗)的紧急使用授权,这也是首款正式上市供人类使用的mRNA疫苗^[129,130]^。基于此,为了进一步提升mRNA疫苗的安全性与有效性,Faro-Viana等^[131]^设计并开发了一种PEG化脂质体mRNA疫苗。该疫苗封装了编码SARS-CoV-19刺突以及糖蛋白的核苷修饰mRNA,以保护感染者免受危及生命的感染。

### 2.3 基于病症部位的药物递送应用

#### 2.3.1 大脑靶向的应用

近年来,尽管研究在中枢神经系统(CNS)疾病和药物递送方面取得了一定的进展,但大脑靶向仍然是一项极具挑战性的任务,特别是针对多发性硬化症、中风以及神经退行性疾病等严重威胁人类健康的CNS疾病^[131,132]^。其中,一个关键因素在于血脑屏障作为一道选择性半透层,限制了药物的渗透性,影响了其正常疗效的发挥。鉴于此,可以将具有不同表面修饰的脂质体作为一种DDS,以实现药物向脑部的有效输送^[133]^。Zhang等^[134]^发明了一种高效的脑部药物递送方法,该方法通过在脂质体表面修饰结合了载脂蛋白的Aβ短肽,并利用血脑屏障上的转运受体,将药物DOX精准靶向递送至脑部,成功实现了脑部药物的有效递送。此外,有研究^[135]^指出,将TF与装载有单克隆抗体的脂质体表面结合,能够提升血脑屏障的渗透率,从而增强脂质体在帕金森病患者脑部的摄取效率。

#### 2.3.2 肺靶向的应用

肺部拥有较大的表面积和薄的上皮层,且血流连续不断,因此利用脂质体将药物靶向输送至肺部,能有效避免口服药物的首过效应。这种方法为呼吸系统疾病(如哮喘、囊性纤维化及慢性阻塞性肺病等)的治疗提供了一种快速的全身给药途径,从而使肺部成为药物输送的有利场所^[136]^。目前,脂质体在临床上已被用于肺部疾病和损伤的治疗。近期,一项研究设计了一个尺寸为100 nm的脂质体,并优化了其脂质成分,主要以二棕榈酰磷脂酰胆碱(DPPC)为主。该脂质体对药物甲强龙(MPS)和化痰药乙酰半胱氨酸(NAC)的包封率分别高达98%和92%。此外,该脂质体能够有效减轻由急性呼吸窘迫综合征(ARDS)引发的肺部损伤^[137]^。此外,Cao等^[138]^开发了一种联合治疗方法,该方法利用可电离脂质体纳米粒(ASNPs),共负载抗氧化药物虾青素(AST)和针对转化生长因子*β*1(TGF-*β*1)的siRNA,以减轻Ⅱ型肺泡上皮细胞(AEC2s)的损伤,同时抑制成纤维细胞的活化和分化,从而增强对特发性肺纤维化(IPF)的治疗效果。总之,脂质体在治疗过程中展现出的卓越疗效已在实验室研究及临床分析中得到了验证,尤其是在肺部疾病的治疗领域中。

#### 2.3.3 肝脏靶向的应用

化疗一直以来是肝癌的主要治疗手段。然而,传统的化疗方法因具有高全身毒性和缺乏特异性而受到限制^[139]^。Tian等^[140]^开发了一种由壳聚糖/PEG-甘草次酸(CTS/PEG-GA)纳米粒子组成的肝脏靶向DDS,其中纳米粒子通过离子凝胶技术制备,利用GA作为靶向配体,盐酸DOX作为抗肿瘤药物。研究通过单光子发射计算机断层扫描(SPECT)评估了这些纳米粒子的生物分布,结果显示,含有GA的脂质体能够有效靶向肝癌细胞。近期,Yang等^[141]^设计了一种以GA为配体、芍药苷(PF)为治疗药的肝靶向DDS,在该DDS中,GA修饰的PF脂质体(GPL)相较于非靶向脂质体,在肝脏中展现出了更强的荧光信号,并且GPL显著延长了PF在血液循环中的滞留时间,进而有助于延长药效。以上实验结果表明,脂质体作为一种安全、高效的载体,能够将药物精准输送至肝脏,是实现肝脏靶向药物递送的理想策略。

#### 2.3.4 肾脏靶向的应用

肾脏作为机体代谢和排泄药物的重要器官,往往成为药物损伤的主要目标。此外,肾功能受损还可能影响药物在肾脏内的分布^[142]^。因此,设计针对肾脏的DDS对于解决上述问题、降低肾毒性以及提升肾脏疾病治疗效率至关重要。脂质体是一种具有潜力的肾脏靶向治疗手段。据文献[143]报道,采用阳离子脂质体并通过逆行注射脂质体/互补DNA复合物,可以有效地转导肾小管细胞,这种方法在改善肾脏疾病状况方面展现出显著效果,如碳酸酐酶Ⅱ缺乏症和单侧输尿管阻塞等。当前,肾间质纤维化被视为多种慢性肾脏疾病进展至终末期肾病的共同路径和主要病理基础^[144,145]^。因此,终止或逆转肾间质纤维化成为阻断慢性肾病进程的新策略。Li等^[146]^构建了一种脂质体递药系统(CREKA-Lip),该CREKA-Lip能够主动靶向至肾间质纤维化的主要病变细胞(肾间质成纤维细胞)。研究结果表明,该CREKA-Lip成功将雷公藤红素靶向递送至纤维化的肾脏区域,不仅增强了其对肾间质纤维化的治疗效果,还有效降低了药物的全身毒副作用。

## 3 外泌体的药物递送应用

药物分布对于保障治疗分子的有效性和安全性具有关键作用。在当代药物管理技术的发展过程中,脂质体、胶束、树枝状聚合物、聚合物纳米颗粒以及无机纳米颗粒等已被广泛用作药物递送工具^[147]^。然而,这些外源性药物载体常伴随多种副作用及不良反应^[147]^。相比之下,外泌体作为内源性、天然的药物递送载体,展现出其独有的优势^[148,149]^。

外泌体是微小的EVs(尺寸为30~100 nm)^[150]^。外泌体不仅能够传递mRNA、miRNA、线粒体DNA和蛋白质等生物分子,而且由于具备脂质双分子膜结构,其还能有效保护所装载的外源性药物免受机体降解。因此,已有许多研究使用外泌体来运载核酸类药物、蛋白质和肽类药物以及小分子化学药物等^[151]^。外泌体在生物医学领域的应用如图2所示。

**图2:**
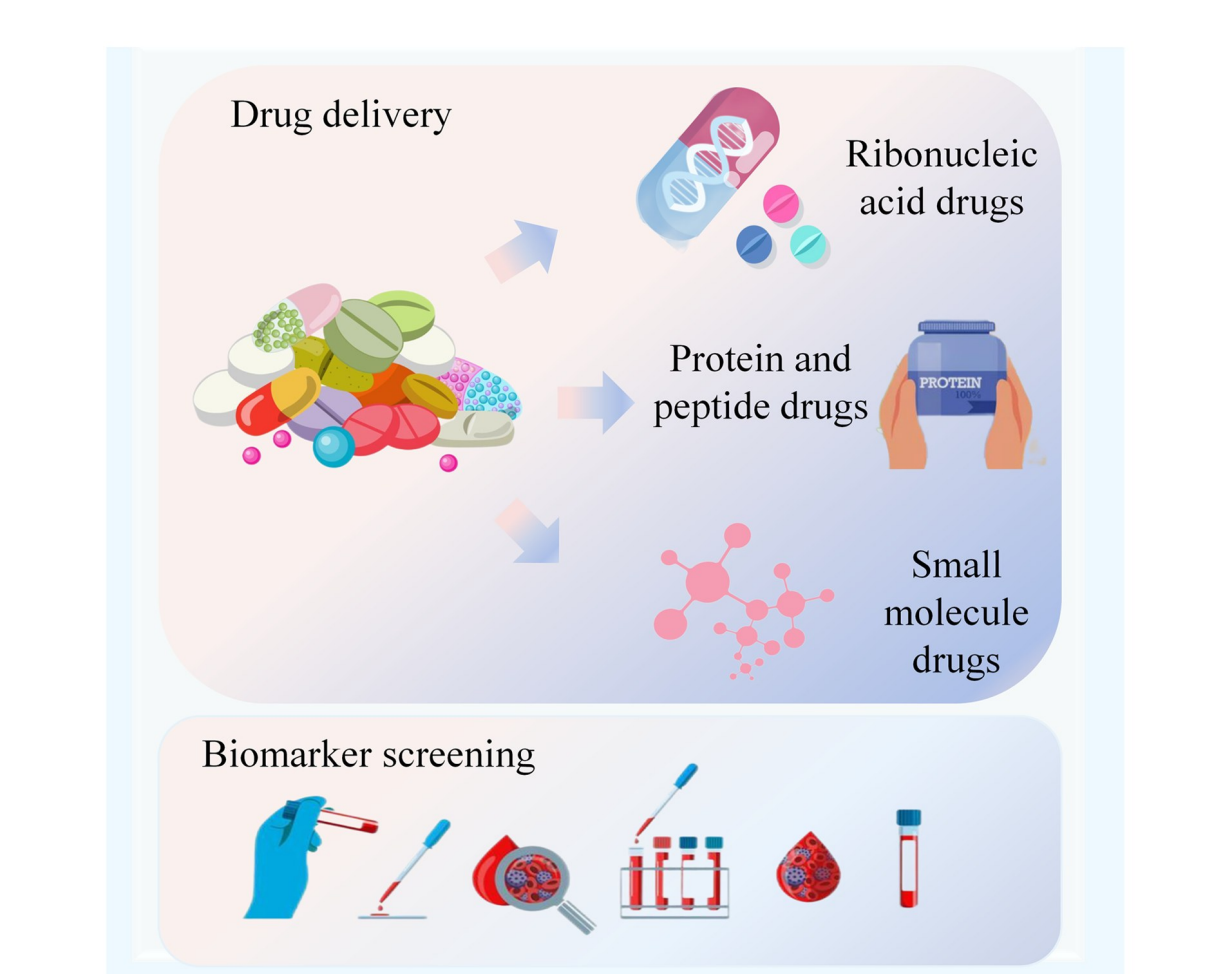
外泌体的生物医学应用

### 3.1 核酸类药物递送

小分子药物通常将蛋白质作为药物靶点,然而人体内有许多“致病蛋白”并不适合作为药物靶点,因此针对这些蛋白的疾病往往缺乏有效的治疗手段^[152]^。相比之下,核酸类药物有望解决上述用药限制^[153]^,但核酸类药物的递送是一个挑战性问题。近年来,随着对外泌体研究的日益深入,科学家们发现外泌体有望成为一种理想的核酸递送系统^[154]^。Lamichhane等^[155]^证实,外泌体能够将通过电穿孔技术装载的外源性DNA转移至受体细胞中,这为外泌体在基因治疗领域的应用提供了重要依据。除了电穿孔技术外,目前已有多种新颖的DNA装载方法被开发出来。Lin等^[156]^通过将外泌体膜的脂质双层与脂质体结合,制备出一种外泌体-脂质体杂合体,该杂合体能够有效封装并递送大型DNA分子(如CRISPR/Cas9质粒),同时降低了脂质体本身的毒性。

与基于DNA的治疗方式相比,基于RNA的治疗方式更具优势。mRNA作为一种关键的中间分子,负责将DNA中的遗传信息传递给核糖体以合成蛋白质,被视为治疗多种疾病的极具潜力的工具^[157⇓-159]^。特别是在COVID-19爆发后,mRNA被迅速用于疫苗开发,已有多种mRNA疫苗被成功研制^[160,161]^。然而,mRNA药物研发过程中面临的主要挑战在于其易受核酸酶降解以及易触发免疫反应^[162]^。在此背景下,外泌体作为一种天然的递送载体,为解决mRNA的有效递送问题提供了新思路。Tsai等^[163]^研究发现,外泌体-脂质体杂合体能够利用Antares2 mRNA有效转染靶细胞,并在此基础上成功研发出多种mRNA COVID-19疫苗。

miRNA是一类高度保守的单链RNA,长度为19~25个核苷酸,一般位于基因组的非编码区^[164]^。由于miRNA广泛参与基因调控过程,且其具有体积小和易于装载的特性,基于外泌体的miRNA疗法得以迅速发展。Rezaei等^[165]^利用肿瘤来源的外泌体递送miR-375模拟物,有效抑制了结肠癌细胞的迁移和侵袭。为了进一步增强抗肿瘤效果,Liang等^[166]^将miR-21i与化疗药物5-氟尿嘧啶(5-FU)共同封装在外泌体内,并通过表皮生长因子(EGFR)受体介导的内吞作用,将这些装载有治疗药物的外泌体靶向递送至对5-FU有耐药性的HCT-116结直肠癌细胞中。研究结果显示,这种联合疗法的抗肿瘤效果得到了显著提升。

siRNA是另一类双链RNA(dsRNA),它能够与目标mRNA完全互补配对,进而引发基因沉默^[167]^。Alvarez-Erviti等^[168]^首次成功使用外泌体递送siRNA,他们通过电穿孔技术将外源性siRNA装载至外泌体中,并进行体外和体内递送,成功敲低了特定基因*BACE1*的表达。因此,可利用装载了siRNA的外泌体来下调癌基因的表达,从而达到抑制细胞增殖和迁移的目的。Alvarez-Erviti等^[168]^还将BCL-2 siRNA装载到自然杀伤(NK)细胞分泌的外泌体中,用于治疗ER+乳腺癌,这一方法显著增强了乳腺癌细胞的凋亡过程。

除了上述核糖核酸外,外泌体还可以递送其他核酸类药物,以用于癌症治疗,如长非编码RNA(lncRNA)、短发夹RNA(shRNA)和适配体等^[169]^。Zheng等^[170]^将携带了编码磷酸酶和张力蛋白同系物假基因1(lncRNA PTENP1)的慢病毒载体转染到人胚肾细胞系293A中,转染后的细胞能够分泌出含有PTENP1的外泌体,这些外泌体通过lncRNA PTENP1与miRNA-17进行竞争性结合,从而调节PTEN蛋白的表达水平。最终,这一过程有效抑制了膀胱癌细胞的恶性行为。数年后,Zheng等^[171]^还采用相同的方法制备了含有细胞间黏附分子1(circLPAR1)的外泌体,这些外泌体通过与真核生物翻译起始因子3h(eIF3h)直接相互作用,阻断了甲基转移酶样3(METTL3)与eIF3h的结合,进而下调了溴结构域蛋白4(BRD4)的表达水平,以此来实现对结直肠癌细胞生长的抑制。综上所述,外泌体作为核酸类药物的递送系统,正在为癌症治疗领域做出重要贡献。

### 3.2 蛋白质和肽类药物递送

与小分子药物相比,蛋白质和多肽类药物展现出高活性、强特异性、低毒性以及明确的生物功能等优势,并且它们适用于多种疾病的治疗,包括肿瘤、糖尿病、自身免疫性疾病、神经系统疾病及心血管疾病等^[172]^。近年来,利用外泌体装载蛋白质和多肽类药物已成为研究热点。Liu等^[173]^开发了一种可用于口服靶向治疗炎症性肠病(IBD)的蛋白递送纳米系统(Gal-IL10-EVs(C/A))。Gal-IL10-EVs(C/A)的生物相容性高,且具有pH响应性药物释放功能和巨噬细胞靶向能力,为生物活性蛋白的口服递送以及肠道疾病的治疗提供了一个有效的平台。然而,外泌体的装载效率易受供体细胞和受体细胞条件的影响,且部分天然外泌体在靶向性方面存在不足。鉴于此,Dooley等^[174]^研究出了两种新型“支架蛋白”(PTGFRN和BASP1),在外泌体供体细胞中,通过将目标蛋白(如细胞因子、抗体片段、RNA结合蛋白、疫苗抗原、Cas9酶和肿瘤坏死因子等)与PTGFRN或BASP1融合表达,可以高效且稳定地将待递送的蛋白质或多肽类药物搭载在外泌体膜的内侧或外侧。这一发现为外泌体的内源性载药提供了一种新颖、高效且稳定的方法。近期,有研究^[175]^报道了一种创新的胞内蛋白递送方法,该方法利用外泌体作为载体,克服了传统外泌体载荷富集技术中必须依赖融合支架的限制,成功实现了多种天然胞内蛋白的高效装载与递送,为蛋白质药物的研发与应用开辟了新的途径。

### 3.3 小分子药物递送

小分子药物具有合成简便、可口服及易于进入细胞等优势^[176]^。与游离小分子药物相比,采用外泌体递送系统的小分子药物展现出更高的生物利用率,且外泌体作为小分子药物的递送载体已成为研究焦点^[177]^。DOX作为一种常用的抗癌药物,广泛应用于多种癌症的治疗,然而长时间接触DOX会导致显著的细胞毒性^[178]^。有研究^[179]^表明,外泌体作为DOX的递送载体,能够提升其治疗指数。通过外泌体递送的DOX在一定程度上阻碍了药物穿透心肌内皮细胞,从而有效降低了心脏毒性。这一发现在乳腺癌和卵巢癌的小鼠肿瘤模型实验中得到了验证。与游离DOX相比,外泌体装载的DOX具有更高的安全性和有效性。为了提高DOX在外泌体中的装载效率,Thakur等^[180]^研发了一种微流体装置,该装置能够有效提升抗癌药物(如DOX和紫杉醇)在外泌体中的装载率,进而增强通过外泌体向胶质瘤细胞(GM)递送药物的效率。此外,有研究团队发现,热应激能够促使含有DOX的肿瘤细胞增加外泌体的分泌数量,并强化这些外泌体对肿瘤细胞的抗肿瘤作用,这为将化疗与热疗相结合的癌症治疗策略提供了新的设计思路^[181]^。

表2汇总了不同类型药物在外泌体中的部分装载方式。

**表2:** 不同类型药物在外泌体中的部分装载方式

Type of drugs	Loading methods	Refs.
Ribonucleic acid drugs	electroporation, convergence technology	[155,156]
Protein and peptide drugs	covalent modification, convergence technology	[173,174]
Small molecule drugs	electroporation, chemical induction	[179,180]

## 4 脂质体与外泌体的生物标志物筛选应用

### 4.1 脂质体的生物标志物筛选应用

基于脂质体的DDS展现出了改善多种疾病治疗效果的巨大潜力^[182]^,然而其临床转化成效还没有达到预期,部分原因是缺乏用于患者分层的生物标志物^[183]^。在肿瘤药物研发中,生物标志物常伴随诊断手段被广泛应用于患者分层。生物标志物筛选与患者分层策略对于应对癌症的高度异质性至关重要,它们能够精确筛选出最可能从分子靶向药物治疗中获益的患者群体。这些分子靶向药物包括激酶抑制剂、治疗性抗体以及抗体-药物偶联物等。因此,生物标志物筛选与患者分层策略在推动这些药物的临床开发与应用中发挥着举足轻重的作用^[184]^。Ito等^[185]^将^111^In标记的脂质体作为生物标志物,并通过SPECT技术观测这些脂质体在肿瘤部位的聚集情况,以此来评估装载有多柔比星的脂质体的抗肿瘤效果。May等^[186]^运用监督机器学习(supervised machine learning)技术对小鼠肿瘤模型中的纳米药物积累数据进行分析,得出了与肿瘤内DOX脂质体含量相关的生物标志物评分。研究还进一步提出,肿瘤血管密度和肿瘤相关巨噬细胞密度可作为肿瘤组织病理学的关键生物标志物,有望用于预测纳米药物在肿瘤部位的积聚情况,以及在临床药物试验中实现对患者的有效分层。此外,生物标志物的检测对于疾病的早期诊断、治疗监测以及预后评估也具有重大意义。Liu等^[187]^开发了一种基于超灵敏磁性生物发光纳米脂质体的新技术。研究以甲胎蛋白(AFP)作为模型蛋白,使用磁性生物发光纳米脂质体进行捕获和分离,并通过便携式ATP光度计对血液中的AFP生物标志物进行即时检测(POCT)。这一技术不仅提升了生物标志物检测的灵敏度和准确性,还凭借其便携性为临床快速诊断及个性化治疗提供了新的思路,为生物标志物的检测开辟了一条新的途径。

此外,脂质体能够通过与配体或刺激响应性化合物结合,实现针对特定受体或生物标志物的主动或被动靶向。有研究发现,作为鉴定生物标志物的工具,脂质体表面的不同修饰会影响其识别的生物标志物数量。例如,通过静电力涂覆多糖(如透明质酸(HA)、海藻酸盐(ALG)、肝素(DXS)、岩藻糖硫酸盐(FUC)和软骨素硫酸盐(CS))的脂质体,能够识别到数量最多的相关生物标志物,这可能是因为多糖与细胞表面蛋白之间存在广泛的相互作用有关。相反,PEG作为抗粘连聚合物,在PEG修饰的脂质体上几乎未能检测到相关的生物标志物,其数量和统计显著性都非常低。这表明PEG修饰减少了脂质体与细胞表面蛋白之间的非特异性相互作用,进而导致可识别的生物标志物数量减少。聚乳酸-羟基乙酸共聚物(PLGA)制剂由于本身特性,其可鉴定的生物标志物数量则与表面修饰基团无关^[188]^。

### 4.2 外泌体的生物标志物筛选应用

外泌体由组织细胞分泌,存在于血液和各种体液中,如外周血、尿液、唾液和脑脊液等^[148]^。外泌体在体内分布广泛,携带了来自多种分泌细胞的生物信息分子,可调节许多病理和生理过程,包括免疫反应、炎症、肿瘤生长和感染等。健康个体与不同疾病的患者会将含有不同含量RNA和蛋白质成分的外泌体释放到血液循环中,这些外泌体可作为生物标志物进行检测^[189]^。与传统的游离血清核酸和蛋白质标志物相比,外泌体具有更强的靶向性、更多的生物信息和更小的检测基质干扰,是生物标志物筛选领域的研究热点^[190]^。

研究表明,癌症衍生的外泌体能够影响癌症在各个阶段的发展。作为新兴的生物标志物来源,外泌体在癌症早期检测中展现出诊断潜力^[191]^。一项针对血浆外泌体miRNA的研究指出,这些分子能够有效区分乳腺癌患者与正常人群^[192]^。Zhai等^[193]^采用了一种核酸功能化的Au nanoflare探针技术,该探针能够直接进入血浆外泌体并特异性地靶向miR-1246,从而触发定量荧光信号,成功实现了血浆外泌体中miR-1246水平的原位检测。利用这项技术,外周血中外泌体miR-1246的原位检测能够以100%的敏感性和93%的特异性准确区分出46例乳腺癌患者与28例健康对照者。Lu等^[194]^利用微流控系统,通过液滴数字聚合酶链式反应技术,实现了外泌体的片上分离以及肺癌相关RNA的分析。该系统能够高效地富集并分析外泌体中的RNA,对于肺癌的早期诊断和治疗监测具有重要的临床意义。此外,在心血管疾病中,受损的内皮细胞会分泌异常的外泌体,这些外泌体可作为心血管疾病诊断的有效生物标志物^[195]^。Zhang等^[196]^研究发现,血清中外泌体的miR-942-5p、miR-149-5p和miR-32-5p可作为稳定性冠状动脉疾病(SCAD)的潜在诊断生物标志物。Zheng等^[197]^报道了一种新型DDS,该DDS将脂质体与外泌体结合,制备了一种基于膜融合技术的混合外泌体(MFHE)。研究结果表明,MFHE可用于疾病诊断,如通过检测血液中的SARS-CoV-2 RNA来诊断COVID-19等。此外,众多研究也表明,外泌体在阿尔茨海默病^[198]^、糖尿病^[199,200]^以及病毒感染^[201]^等其他疾病中同样展现出了良好的生物标志物筛选功能。

随着临床诊断需求的日益增长,外泌体作为诊断生物标志物的应用正呈现商业化的趋势^[202]^。一项研究对比分析了4种常用的外泌体miRNA商用试剂盒(ExoQuick^TM^外泌体沉淀溶液(EXQ)试剂盒、血清或血浆总外泌体分离(TEI)试剂盒、exoRNeasy血清/血浆Midi(EXR)试剂盒和RIBO^TM^外泌体分离试剂(REI))的优缺点,并探讨了它们在血清和血浆样本中的应用效果。结果发现,EXQ试剂盒能够获得相对较高的外泌体产量。然而,这4种试剂盒在提取过程中普遍存在白蛋白杂质的问题。相比之下,采用TEI试剂盒能够获得更为纯净的分离物。在特定外泌体miRNA的回收率方面,EXR和EXQ试剂盒表现更为出色^[203]^。由此可见,外泌体miRNA商用试剂盒能够为临床诊断提供有力辅助,进一步推动外泌体在临床领域的广泛应用。

## 5 展望

脂质体作为一种灵活且功能多样的纳米级DDS,受到了广泛的关注与研究。外泌体是一种EVs,参与细胞间通信,同样成为研究的热点。这两种囊泡在癌症治疗、基因治疗及疫苗接种领域展现出巨大的潜力。作为DDS,它们克服了传统游离药物递送的局限及耐药性问题。然而,随着研究的深入,这两种囊泡也面临着诸多挑战。脂质体药物递送的研究需要持续提升设计与制备水平、加强毒理学评估、细胞相互作用研究以及临床评估能力。对于外泌体,由于其几乎能被所有细胞分泌,缺乏特异性生物标志物来有效分离和鉴定病变组织,且其细胞内分选机制尚未完全明了,这限制了其在临床和用药方面的应用。然而,有理由相信,随着脂质体和外泌体基础研究和纯化检测技术等的不断发展,它们将在临床诊断乃至更多新兴领域中得到更加广泛的应用。
